# Selection of Suitable Endogenous Reference Genes for Relative Copy Number Detection in Sugarcane

**DOI:** 10.3390/ijms15058846

**Published:** 2014-05-19

**Authors:** Bantong Xue, Jinlong Guo, Youxiong Que, Zhiwei Fu, Luguang Wu, Liping Xu

**Affiliations:** 1 Key Laboratory of Sugarcane Biology and Genetic Breeding, Fujian Agriculture and Forestry University, Ministry of Agriculture, Fuzhou 350002, China; E-Mails: xuebantong@163.com (B.X.); jl.guo@163.com (J.G.); queyouxiong@hotmail.com (Y.Q.); fuzhiwei1991@126.com (Z.F.); 2 School of Agriculture and Food Sciences, University of Queensland, Brisbane, QLD 4072, Australia; E-Mail: l.wu@uq.edu.au

**Keywords:** sugarcane, endogenous reference gene, absolute quantification, copy number

## Abstract

Transgene copy number has a great impact on the expression level and stability of exogenous gene in transgenic plants. Proper selection of endogenous reference genes is necessary for detection of genetic components in genetically modification (GM) crops by quantitative real-time PCR (qPCR) or by qualitative PCR approach, especially in sugarcane with polyploid and aneuploid genomic structure. qPCR technique has been widely accepted as an accurate, time-saving method on determination of copy numbers in transgenic plants and on detection of genetically modified plants to meet the regulatory and legislative requirement. In this study, to find a suitable endogenous reference gene and its real-time PCR assay for sugarcane (*Saccharum* spp. hybrids) DNA content quantification, we evaluated a set of potential “single copy” genes including *P4H*, *APRT*, *ENOL*, *CYC*, *TST* and *PRR*, through qualitative PCR and absolute quantitative PCR. Based on copy number comparisons among different sugarcane genotypes, including five *S. officinarum*, one *S. spontaneum* and two *S.* spp. hybrids, these endogenous genes fell into three groups: ENOL-3—high copy number group, TST-1 and PRR-1—medium copy number group, P4H-1, APRT-2 and CYC-2—low copy number group. Among these tested genes, *P4H*, *APRT* and *CYC* were the most stable, while *ENOL* and *TST* were the least stable across different sugarcane genotypes. Therefore, three primer pairs of P4H-3, APRT-2 and CYC-2 were then selected as the suitable reference gene primer pairs for sugarcane. The test of multi-target reference genes revealed that the *APRT* gene was a specific amplicon, suggesting this gene is the most suitable to be used as an endogenous reference target for sugarcane DNA content quantification. These results should be helpful for establishing accurate and reliable qualitative and quantitative PCR analysis of GM sugarcane.

## 1. Introduction

Sugarcane (*Saccharum* spp. hybrids) is the world’s largest crop, accounting for 80% of all sugar produced in the world, cultivated in 101 countries and the cultivated area was about 26.1 million hectares in 2012 according to the FAO estimates, with a worldwide harvest of 1.83 billion tons. Sugarcane, accumulating large quantities of sucrose in stem tissues, is one of the most important sugar crops and has also been developed into an important energy crop [1]. It has proved very difficult to obtain a multi-merits sugarcane variety with high biomass, high sugar content and excellent disease and pest resistance by relying solely on traditional hybridization using asexually reproduced modern sugarcane varieties, which is a complex of *S. officinarum* (chromosome number 80), *S. spontaneum* (chromosome number from 40 to 128) and even containing *Erianthus arundinaceum* in sugarcane clones bred in recent five years in China, with a complex allopolyploid and aneuploid genetic background [2]. Genetic transformation has a potential to introduce desirable traits into target crops and to supplement traditional plant breeding techniques [3], resulting in a revolution in sugarcane breeding and sucrose production [4]. Sugarcane has several advantages that make it an ideal candidate for improvement via genetic engineering. It is rarely flowering and producing fewer viable seeds from commercial sugarcane cultivars especially in the commercial cultivation, which greatly reduces the potential of genetic drifting from genetically modified (GM) sugarcane. Unlike other GM food crops, GM sugarcane could be easily accepted by the public and regulatory authorities, as refined white sugar is the most chemically pure food derived from plants and has been found to be free from DNA and proteins expressed from the introduced transgene [5]. Along with the first sugarcane transgene event released for commercial cultivation in 2013, the establishment of detection technology for transgenic components of sugarcane source has become necessary and urgent. When referring to transgene breeding, the agronomic and economic characters of transgenic sugarcane can be affected by a number of factors, including but not confined to the site of insertion, the direction of adjacent transgenic elements and gene copy number. Higher copies in transgenic sugarcane can even cause co-suppression, mainly from transcriptional gene silencing (TGS) or post-transcriptional gene silencing (PTGS) [6,7]. *Agrobacterium*-mediated and particle bombardment are the two methods widely used in sugarcane genetic transformation [8,9]. However, *Agrobacterium-*mediated transformation is not as successful as that by gene gun bombardment in sugarcane. Consequently most transgenic sugarcane events have been produced by the latter technique and tend to show high copy numbers of recombinant inserts [10]. Besides, even though fewer copies of recombinant inserts are obtained in transgenic sugarcane via *Agrobacterium*-mediated transformation, it may still contain two or more copies of the recombinant inserts. To accelerate the application of transformation in sugarcane, it is necessary and urgent to identify endogenous reference genes for transgenic sugarcane detection.

Different molecular techniques such as Southern blotting [10], multiplex probe amplification and hybridization and microarray analysis [11] have been used to explore the gene copy number in transgenic plants. Although routinely applied and reliable, these methods are labor-intensive, time-consuming, and require considerable amounts of DNA. In addition, they produce inaccurate estimation of the foreign gene copy number and may involve the use of hazardous radioisotopes. The abovementioned disadvantages rank these methods as impractical on a large scale screening of transgenic plants in early stages [3,11,12]. Moreover, Southern blotting does not accurately reflect the presence of rearranged copy numbers when relevant restriction sites are lacking [13]. These techniques are even harder in the sugarcane hybrid due to the complicated genome of 10 to 12 Gb [14] and 3.05–8.91 pg/2C [15]. In addition, sugarcane transgenic breeding needs a transformation population for selection of plants with the ideal phenotypes. Therefore, the method with high-throughput and with the capability to estimate high transgene copy numbers should be an ideal complementary to the other methods such as Southern blotting.

To overcome those limitations, a fast, sensitive and effective method has been developed for estimating transgene copy number by quantitative real-time PCR technique (qPCR) [12,16–18]. This method can trace the amplification of a target DNA sequence by monitoring fluorescence emitted from specific double-stranded DNA binding dyes or the fluorophore-labeled during the process of reaction [3,12,19]. In addition, it is valuable for the detection of rearrangements between two transgenic plant lines. There are mainly two kinds of qPCR assays in use: relative quantification [15,20] and absolute quantification [21]. Though both approaches were developed for relative copy number estimates, the former is based on a ratio between two targets while the latter quantifies an unknown amount of target towards a standard curve for the same target [22,23]. So far, qPCR has been employed to detect transgene copy number in several plant species, such as *Nicotiana tabacum* [24], *Brassica napus* [25], *Zea mays* hybrids [20], *Oryza sativa* spp. [26], *Gossypium* spp. [27] and *Solanum lycopersicum* [15].

A sensitive and reliable endogenous reference gene is essential for species differentiation, and for detection of genetically modified organism (GMO) products in transgenic plants [28]. The endogenous reference genes should be stable among cultivars and show a low copy number (ideally but not indispensable single copy) in the haploid genome [16], species-specific due to the need to determine the species source of the detection samples, especially when the detection samples are plant processed product, such as food. For sugarcane, the “single copy” means “a gene that exists as a single copy per haploid genome”. More specifically, due to the estimated ploidy (8) of sugarcane, single cell copy number of a certain candidate gene should be divided by 8, thus, a future value less than 1 means the target gene detected presents a single copy per haploid genome. To date, the corresponding endogenous reference genes have been developed in several crops, such as *Lectin* and *β-actin* for *Glycine max* [29], *hmga* gene for *Z. mays* [30], *PEP* [31] and *HMGI/Y* [32] for *B. napus*, *LAT52* for *Lycopersicon esculentum* [33] and *SPS* for *O. saliva* [31]. However, the increased number of reported endogenous reference genes has made it difficult to select the best candidate for a specific GMO analysis, and how to harmonize these endogenous reference genes is becoming not only important but also necessary in some cases. To our knowledge, there is only the publication by Casu *et al.* [1] focused on endogenous reference genes for sugarcane. It relies on the reference gene sequences derived from *Sorghum bicolor*, suggesting that there is still a lack of relatively stable endogenous reference genes for detection of transgenic products as an internal positive control in sugarcane, and highlighting the necessity of selection of endogenous reference genes for transgenic sugarcane detection.

To find out low copy genes with small copy number variation among different sugarcane genotypes, six potential endogenous genes, *P4H*, *APRT*, *ENOL*, *CYC*, *TST* and *PRR*, were assessed in the present study for their suitability in qPCR. After preliminary validation of the copy numbers of the above genes, those appearing to be low copy numbers were selected for construction of the vector to obtain a multi-target reference plasmid for further test in the same background. The results presented in this paper will help further efforts to quantify DNA content or copy number, contributing to the advance of sugarcane genetic engineering and its commercial application.

## 2. Results

### 2.1. Assessment of Primer Within-Species-Specificity

#### 2.1.1. Assessment of Primer Within-Species-Specificity by PCR

**Figure 1.**
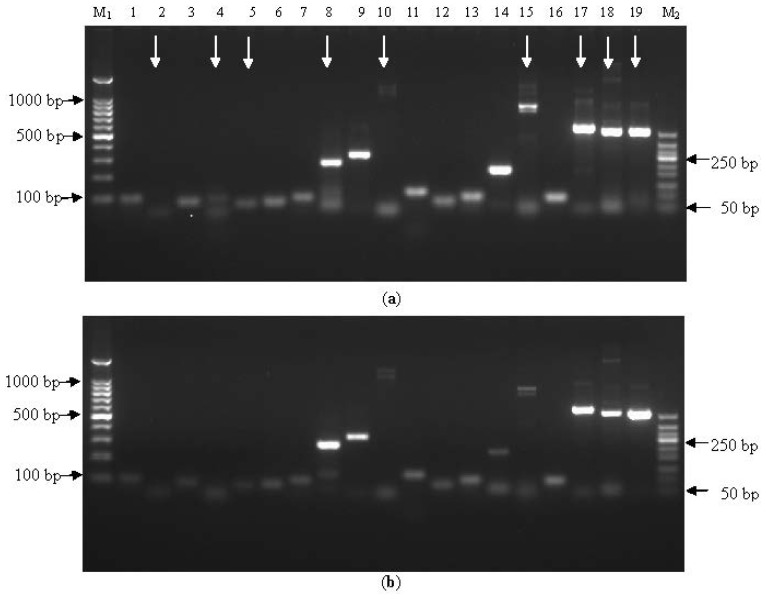
PCR amplification products of potential reference genes. (**a**) ROC22; (**b**) Badila; M_1_ 100 bp Marker, 1 P4H-1, 2 P4H-2, 3 P4H-3, 4 ENOL-1, 5 ENOL-2, 6 ENOL-3, 7 TST-1, 8 TST-2, 9 TST-3, 10 TST-4, 11 APRT-1, 12 APRT-2, 13 CYC-1, 14 CYC-2, 15 CYC-3, 16 PRR-1, 17 PRR-2, 18 PRR-3, 19 PRR-4, M_2_ 50 bp Marker, white arrows indicated the nine potential reference primer pairs which were unsuitable for further evaluation. On that gel an arrow would have been added to lane 14 but with other shown gels are not shown, therefore it was also selected.

Nineteen specific primer pairs were designed for six candidate reference genes (Table 1), each of which was tested for single fragment gene amplification in the preliminary experiments of PCR, using the genomic DNA, isolated from sugarcane cultivar ROC22 and Badila, as templates. Ideal primer pairs were selected if their use through PCR resulted in a single band present on the gel for both ROC22 and Badila with clear background, indicating that the primers were specific to that gene only. After gel electrophoresis, gel purification, sequencing and alignment of the amplified fragments, a total of ten primer pairs were selected based on the above selection criterion for subsequent qPCR. Experiments were conducted as: P4H-1 and P4H-3 corresponding to the gene *P4H*, ENOL-3 to the gene *ENOL*, TST-1 and TST-3 to the gene *TST*, APRT-1 and APRT-2 to the gene *APRT*, CYC-1 and CYC-2 to the gene *CYC* and PRR-1 to the gene *PRR*, respectively (Table 1, Figure 1).

#### 2.1.2. Assessment of Primer Performance by qPCR

Standard curves of the above ten selected primer pairs were established respectively to evaluate the amplification efficiency, and melting curves were used to check the within-species-specificity of each qPCR reactions. An ideal reaction reaches an *E* value close to 1.0, representing a PCR efficiency of 100% [19,26–28]. The data in Table 2 indicated that the *R*^2^ values of the nine primer sets for the standard curves were >0.989 and the estimated amplification efficiencies (*E*) were between 0.926 and 1.200, except for the primer set of CYC-1 (Table 2). A single sharp peak in each of the dissociation curves, which corresponds to each of the primer sets, indicated a single melting event, and thus represents a single amplification product, but one which might arise from repeats within the sugarcane genome. In this context, the primer pairs P4H-3, APRT-2, CYC-2 and TST-1 were superior to P4H-1, APRT-1, CYC-1 and TST-3, respectively, based on their melting curves (Supplementary Figure S1). The same results were also verified on the sugarcane cultivars *Saccharum* spp. hybrids ROC22 (data not shown). These observations resulted in the primer pairs P4H-3, APRT-2, CYC-2, TST-1, ENOL-3 and PRR-1 being selected for the development of the qPCR assay.

### 2.2. Screening for Low Copy Genes

#### 2.2.1. Copy Number Calculation of the Candidate Reference Genes

Copy number calculation of the candidate reference genes in the genomic DNA of ROC22 and Badila were calculated according to the formula 4.10. Recently, estimated genome sizes of 10 Gb for ROC22 and of 7.88 Gb for Badila were reported [34], though the chromosome number and genome size are likely to be quite variable. Based on this assumption, the copy number calculation formula for ROC22 is Copies/genome = 10*^X^*^t^/[25 × 10^−9^ g × 6.02 × 10^23^/(10,000 (M bp) × 10^6^ × 660)], while the formula for Badila is Copies/genome = 10*^X^*^t^/[25 × 10^−9^ g × 6.02 × 10^23^/(7880 (M bp) × 10^6^ × 660)]. Then, an estimated copy number was generated for each gene (Table 3). The copy number of these endogenous genes fell into three groups: a high copy number group containing ENOL-3, a medium copy number group including TST-1 and PRR-1, and a low copy number group consist of P4H-3, APRT-2 and CYC-2. The experimental data demonstrated that, in these two varieties, the most stable reference genes were *P4H*, *APRT* and *CYC* because of the closer copy number, while the least stable genes were *ENOL* and *TST*, especially the primer pair ENOL-3 (Table 3). Moreover, three genes *P4H*, *APRT* and *CYC* showed no significant difference in the genomic DNA of ROC22. Similarly, these three genes were not significantly different in Badila. According to these results, primer pairs P4H-3, APRT-2 and CYC-2 were primarily selected as the suitable reference gene primer pairs for further testing.

#### 2.2.2. Stability Analysis of the Selected Endogenous Reference Genes in Different Sugarcane Varieties

Due to the exact genome size and ploidy of modern sugarcane being unknown, it is necessary to verify the stability of selected primers P4H-3, APRT-2 and CYC-2 in more sugarcane genotypes with a different genetic background. Considering the ploidy and chromosome composition of *S. officinarum* and *S. spontaneum* are much clearer than modern sugarcane (*S*. spp. hybrids), one *S. spontaneum* SES208 and several different genotypes of *S. officinarum* including Black Cheribon (Yunnan, China), Black Cheribon (Fujian, China), Loethers, LA Purple and Crystalina were chosen for further testing. In addition, modern sugarcane varieties Q117 and YCE05-179 were included, of which Q117 was one of the sugarcane varieties used in the study by Casu *et al.* (2012) and YCE05-179 was a newly derived sugarcane line with outstanding yield-related traits and disease resistance. The estimated copy numbers for these sugarcane genotypes were listed in Table 4. Results of qPCR from different sugarcane genotypes further proved the endogenous genes *P4H*, *APRT* and *CYC* were sugarcane-specific in low copy number through several sugarcane cultivars.

#### 2.2.3. Evaluation of Three Candidate Genes in a Multi-Target Reference Plasmid

In order to evaluate the three candidate endogenous reference genes *P4H*, *APRT* and *CYC*, in a uniform background as well as to overcome the problem of suitable positive plant material and avoid differences of DNA extraction, a multi-target reference plasmid, carrying the primer sequences (P4H-3, APRT-2 and CYC-2) corresponding to the above three genes, was constructed and assigned as pMD18-PAC with a size of 3005 bp. The three standard curves, which were suitable for analyzing the dosages of *P4H*, *APRT* and *CYC* genes, were established individually based on the 10-fold dilution with a dosage range of 1.0 × 10^6^–1.0 × 10^2^ copies per μL. The estimated copy numbers of *P4H*, *APRT* and *CYC* genes were about 3, 2 and 4 in *S*. spp. hybrid ROC22, and were 2, 2 and 3 in *S. officinarum* Badila, respectively. Using *APRT* gene as the internal control, the relative abundance of *P4H*/*APRT*, *APRT*/*APRT* and *CYC*/*APRT* were 1.5, 1.0 and 2.0 in ROC22, respectively. Similarly, the corresponding relative abundance values present in *S. officinarum* Badila were 1.0, 1.0 and 1.5, respectively (Table 5).

For the technical performance of qPCR, the mean squares of coefficient of determination (*R*^2^) were close to 1.0 for all three genes *P4H*, *APRT* and *CYC* (Table 5), indicating a good linearity between the initial copy numbers and the fluorescence values (*C*t). The PCR efficiencies (*E*) of *P4H*, *APRT* and *CYC* were all also close to 1.0 (Table 5). Apparently, these three standard curves corresponding to *P4H*, *APRT* and *CYC* genes established in this study were applicable to quantify the gene dosages in sugarcane (*S.* spp. hybrids, *S. officinarum* and *S. spontaneum*). At the same time, both *P4H* and *APRT* genes showed even lower copies or less dosages compared to *CYC* in the genomes of ROC22 and Badila, while the relative abundance values of *P4H* and *CYC* were higher than that of *APRT*. These results indicated that the *APRT* gene was a specific amplicon within the detection using the primer pairs APRT-2, and thus this gene was assumed to be the most suitable one as an endogenous reference gene in sugarcane.

## 3. Discussion

The innovative points in this study are: Firstly, an endogenous reference gene *APRT* was evidenced as the best one in copy numbers detection across a range of eight sugarcane genotypes including *S.* spp. hybrids, *S. officinarum* and *S. spontaneum*; Secondly, the primers designed for qPCR are sugarcane-specific (universal in sugarcane genotypes, as the first point above), as all the primers were designed from sugarcane ESTs; Thirdly, a practically useful plasmid was constructed with multi-target genes for qPCR, which resulted in the detection under the same background. Therefore, this study should serve as a solid foundation for establishing an efficient technique to estimate copy numbers of target genes in sugarcane.

Since the first GM plant, an antibiotic-resistant tobacco, was produced in 1982 [35], an increasing number of transgenic plants, including transgenic sugarcane, have been created and produced with different desirable traits, such as insect resistance, disease resistance and herbicide resistance [36]. A series of GM sugarcane events were reported, including Basta herbicide resistance sugarcane [37], *ScMV-CP* transgenic sugarcane [38], *GAN* transgenic sugarcane [39], *Hs* 1 *pro*-1 of nematode-resistant transgenic sugarcane [40], and *Bt* transgenic sugarcane [41]. In addition, the first transgenic sugarcane event aimed at improvement of drought resistance was released for commercial cultivation in Indonesia in 2013. Today, it is widely adopted that GM sugarcane is on the way of commercialization in more countries. However, there is still a lack of endogenous reference genes available for transgenic sugarcane detection.

The advances in PCR instrumentation and fluorescence chemistry have made it possible to precisely quantify the specific amplification products [12]. Compared to Southern blotting, multiplex probe amplification and microarray analysis, qPCR can analyze hundreds of samples with a small amount of DNA, and significantly expedite the identification of the single copy or low copies insertion at much earlier stages [3]. Due to its simplicity, sensitivity and specificity, the qPCR assay has been widely used in copy number detection [15,24–27]. To make PCR assay more precise and reliable, and to detect genetically modified organism products as an internal positive control, application of appropriate endogenous reference gene is indispensible [42]. Although a series of endogenous reference genes were developed for diploid plants and/or used for TaqMan probes [43,44], only one case study was found on the assessment of transgene copy number in sugarcane by qPCR [1]. In the present study, we evaluated a set of potential “single copy” genes including *P4H*, *APRT*, *ENOL*, *CYC*, *TST* and *PRR*, for their suitability as endogenous reference genes for transgenic detection in sugarcane. We began on the basis of preliminary estimations, by comparing copy numbers of the above six genes among eight different sugarcane genotypes. We then selected the genes with low copy numbers to construct a plasmid with multi-target reference genes for further tests. With the development of new GM crops, more and more reference plasmids have been employed simultaneously to detect multiple foreign genes [16]. Based on the standard curve which was created with a confirmable size multi-target plasmid, qPCR has been used successfully for determining the mass of gDNA that correspond to copy numbers of target sequences in GMO [45,46]. The qPCR assay established here was specifically designed for sugarcane because the primers in qPCR were designed according to sugarcane ESTs. Moreover, qPCR is currently the most sensitive method and the formula used is exponential, so slight differences can result in a wide bias. The value of endogenous reference genes’ copy number in this paper was usually estimated in a range. Thus, increasing the biological and technical replicates is more important for the repeatability of results. More importantly, the selected endogenous reference gene *APRT*, with the feature of low copies, can be considered as a reliable reference one among various sugarcane genotypes as the primer pairs APRT-2 has been tested in several sugarcane varieties with different genetic background.

## 4. Experimental Section

### 4.1. Tissue Samples

Leaf samples of sugarcane cultivars *Saccharum* spp. hybrids ROC22 and YCE05-179, *S. officinarum* Badila and LA Purple and *S. spontaneum* SES208 were collected from the Key Laboratory of Sugarcane Biology and Genetic Breeding of the Ministry of Agriculture; Leaf samples of sugarcane variety Q117, *S. officinarum* Black Cheribon (originated from Yunnan, China), Black Cheribon (originated from Fujian, China), Loethers and Crystalina were provided by the National Sugarcane Germplasm Nursery, Yunnan, China.

### 4.2. Reagents and Consumables

SYBR Green Master (ROX) was purchased from Roche (Shanghai, China); Plasmid Mini Kit and Gel Extraction Kit from Omega Bio-Tek (Shanghai, China); pMD 18-T vector and Ex *Taq* from TaKaRa Biotechnology Co., Ltd., (Dalian, China); competent cell DH5α and DNA Markers from TianGen Biotechnology Co., Ltd., (Beijing, China).

PCR Tubes, PCR Strip Tubes, Optical 96-Well Reaction Plate and other PCR related consumables were from Applied Biosystems (Foster, CA, USA) unless specified.

### 4.3. Extraction and Isolation of Genomic DNA

Genomic DNA was extracted according to the CTAB-based protocol described by Paterson *et al.* [47]. DNA concentrations were measured using a NanoDrop spectrophotometer (Wilmington, DE, USA) and its quality was verified through electrophoresis in 1.0% agarose gels on a Amersham Pharmacia EPS301 electrophoresis apparatus (Little Chalfont, Amersham, UK), stained with ethidium bromide, detected on a Bio-Rad Gel imaging system (Hercules, Contra Costa, CA, USA); The quantified DNA was diluted with deionized water to 100 ng/μL. All DNA samples were stored at −20 °C.

### 4.4. Primer Design for Candidate Endogenous Reference Genes

Six primer pairs from putative reference genes, *P4H*, *APRT*, *ENOL*, *CYC*, *TST* and *PRR*, with source DNA sequences from *Sorghum bicolor* reported by Casu *et al.* [1], were selected as candidate endogenous reference genes in this study. After alignments to sorghum gene sequences, the corresponding fragments were identified from the sugarcane ESTs by BLASTP application (National Center for Biotechnology Information, Bethesda, MD, USA), an additional 13 specific primer pairs targeted at these six putative reference genes were redesigned according to the sugarcane ESTs by Primer Premier 5 software (PREMIER Biosoft International, Palo Alto, CA, USA). The primers used in this experiment are shown in Table 1.

### 4.5. Polymerase Chain Reaction (PCR)

DNA fragments were amplified by PCR based on 25 ng genomic DNA of sugarcane cultivar ROC22 or Badila. A final volume of 25 μL was used, containing 2.5 μL 10× PCR Buffer, 2.0 μL (2.5 mM) of dNTPs, 0.125 μL (5 U/μL) Ex *Taq* polymerase, 1.0 μL (10 μmol/L) of each gene-specific forward and reverse primer and 17.375 μL ddH_2_O. PCR was performed under the following conditions: 98 °C for 30 s (1 cycle); 98 °C for 15 s, 60 °C for 15 s; 72 °C for 15 s (35 cycles). All PCR products were separated by electrophoresis on a 3.0% agarose gel with an Amersham Pharmacia EPS301 electrophoresis apparatus (Little Chalfont, Amersham, UK) in 1× TAE buffer. A Bio-Rad Gel imaging system (Hercules, Contra Costa, CA, USA) was used for observation of the electrophoresis products.

### 4.6. Preparation of Recombinant Plasmid

PCR-amplified fragments were purified with a gel purification kit from Omega Bio-Tek (Shanghai, China) and cloned into the pMD18-T vector from TaKaRa Biotechnology Co., Ltd. (Dalian, China), according to the manufacturer’s instructions. The recombinant plasmid was transformed into competent *E. coli* (*DH5α*) and 100 μL of transformed culture was spread onto LB plates containing ampicillin (50 μg/mL), X-gal (20 mg/mL) and IPTG (50 mg/mL). Transformed (white) colonies were picked up and processed for plasmid isolation and sequencing. Plasmids were extracted using a Plasmid Omega Bio-Tek Mini kit (Shanghai, China) according to the manufacturer’s instructions.

### 4.7. Calculation of Plasmid Copy Number

Plasmid DNA concentration was estimated by measuring the absorbance at 260 nm as described above. Plasmid copy number was calculated according to the following formula:

(1) $$\begin{array}{c} \text{Number of}\;\text{copies }(\text{copies}/\mu \text{L})=6.02\times 10^{23}\;(\text{copies}/\text{mol})\times \text{plasmid concentrations }(\text{g}/\mu \text{L})/\\ {}[\text{number of bases pairs}\times (\text{A}\times 313.2+\text{C}\times 289.2+\text{G}\times 329.2+\text{T}\times 304.2)\;\text{Daltons}]. \end{array}$$[48,49]

### 4.8. Real-Time Quantitative PCR

All qPCR assays were performed on an ABI PRISM 7500 Sequence Detection System (Applied Biosystems, Foster City, CA, USA) in 25 μL reaction volume containing 12.5 μL of 2× SYBR Green PCR Master mix (Roche, Shanghai, China), 1.0 μL of diluted genomic DNA (25 ng) and 1.0 μL (10 μmol/L) each of a gene-specific forward and reverse primer. The following standard PCR reaction conditions were used for all transcripts: 50 °C 2 min; 95 °C 10 min; 45 cycles of 95 °C 15 s, 60 °C 1 min; 1 cycle of 95 °C 15 s, 60 °C 15 s, 95 °C 15 s. The last cycle provided dissociation curves for each sample, allowing for assessment of the specificity of amplification. For each sample test, each PCR reaction had three replicates and the experiment was repeated three times.

### 4.9. Establishment of Standard Curve

Each of the purified plasmid was diluted with sterile deionized water to obtain a standard series from 1.0 × 10^8^ to 1.0 × 10^1^ copies/μL with each step differing by 10-fold. It’s necessary to suspend well by pipetting 30 times when diluting. Assays of qPCR were performed using 25 ng/μL (working concentration) DNA and water as control, with three replicates. After reaction, the values of threshold cycles are achieved. Each standard curve is established by plotting the threshold cycle (*C*t) on the *Y*-axis and the natural log of concentration (copies/μL) on the *X*-axis, and the equation *y* = *k* × *x* + *b*, coefficient of determination (*R*^2^) and percentage of variance in copy numbers were achieved.

### 4.10. Copy Number Calculation of Endogenous Reference Genes in Sugarcane

The copy number for putative reference gene in sugarcane genome DNA can be calculated against its established standard curve. After reaction, the values of threshold cycles are achieved. From the slope of a standard curve, PCR amplification efficiency (*E*) can be calculated according to the equation as follow [50]:

(2) $$E=10{\;}^{\left(-1/\text{slope}\right)}-1$$

The total copy number (10 *^X^*^t^) of each endogenous reference gene is calculated by relating the *C*t value (*Y*_t_) to its corresponding standard curve, then the single cell copy number (*n*) of each endogenous reference gene in the sugarcane samples can be calculated by the following formula:

(3) $$\text{copies }(n)/\text{genome}=10{\;}^{X\text{t}}/[25\times 10^{-9}\;\text{g}\times 6.02\times 10^{23}/(\text{genome size of single cell }(\text{M bp})\times 10^{6}\times 660)].$$

Notes: 25 × 10^−9^ g is the amount of DNA template (measured by UV absorption) used in each qPCR reaction system, 6.02 × 10^23^ is Avogadro’s constant, the genome size of sugarcane can be determined by flow cytometry [51], and average molecular weight (*M*_W_) of a DNA base pair is 660 daltons.

### 4.11. Construction of Multi-Target Reference Plasmid for Screening Low Copy Genes

In order to create a reference plasmid containing all the test sequences corresponding to the putative reference genes for further test in the same background, all the tested sequences were synthesized by Sangon Biotech Co., Ltd. (Shanghai, China) and constructed onto the same vector to obtain a multi-target reference plasmid [45]. The standard curve for putative low copy gene sequences is established based on 1.0 × 10^6^ to 1.0 × 10^2^ copies of reference plasmids, and the validated primer pairs in the above experiments were employed for qPCR with templates of 25 ng/μL sugarcane genomic DNA of ROC22 and Badila. In addition, one of the above genes was used as the internal control to calculate the relative abundance values to compare the gene dosage [46]. The relative abundance values of the putative low copy genes were calculated: relative abundance = (copy number of determined genes in sample DNA/copy number of the internal control gene in sample DNA).

## 5. Conclusions

The present study succeeded in selection of endogenous reference genes for transgenic sugarcane detection. The *APRT*, as an endogenous reference gene, was identified from a set of six putative potential “single copy” ones, *P4H*, *APRT*, *ENOL*, *CYC*, *TST* and *PRR* in sugarcane. The primer pair APRT-2, corresponding to the *APRT* gene, was screened out from nineteen initial primer pairs that corresponded to the above six reference genes. The selected gene of *APRT* was considered as “single copy” based on: (1) low heterogeneity among different sugarcane species; and (2) low copy number. This conclusion was derived from testing on all eight sugarcane genotypes with different genetic background including *S. officinarum*, *S. spontaneum* and the modern varieties (*S.* spp. hybrids). In addition, the copy number was calculated based on the comparison to a multi-target gene plasmid standard curve. Therefore, *APRT* can be used as a reliable endogenous reference gene; when combined with the developed primer pair, it is suitable for transgenic sugarcane precise detection based on absolute quantification using qPCR, which hopefully will accelerate sugarcane genetic engineering and the commercialization of GM sugarcane.

## Supplementary Information



## Figures and Tables

**Table 1.** Primers used in the present study.

Gene	Primer pair #	Primer sequence	Product size/bp	Source sequence ID
*P4H*	P4H-1*	F: 5′-GCGACATCAGAACAGTGTGAA-3′ R: 5′-TTGTACTCTCCGCGGTTTCT-3′	100	sb01g007280*
	P4H-2	F: 5′-GTCCGTAATCCCATACCAGATTTT-3′ R: 5′-CACACTGTTCTGATGTCGCAAA-3′	80	CA107003, CA106927, CA209790, CA131236
	P4H-3	F: 5′-GTGAAAATATAGTAAAAACTGCGAAGGA-3′ R: 5′-TTGTACTCTCCGCGGTTTCTC-3′	84	
*ENOL*	ENOL-1*	F: 5′-TCCTTACAAAGGATGGGAGC-3′ R: 5′-TGTACAGATCACCCAGACGC-3′	96	sb02g023480*
	ENOL-2	F: 5′-TTTTGATCAGGATGACTGGAGTTC-3′ R: 5′-AAATCATCACCCACAATTTGGAT-3′	75	CA229198, CA131106
	ENOL-3	F: 5′-GGACCCTTTTGATCAGGATGAC-3′ R: 5′-AATCATCACCCACAATTTGGATATC-3′	80	
*TST*	TST-1*	F: 5′-ACATGCTGCCATCTGAAAAG-3′ R: 5′-CAGCCCCTTTCCATCATAAA-3′	95	sb08g020860*
	TST-2	F: 5′-GCTGTTCAGTGCTGCTCGTGTT-3′ R: 5′-GGCAAACCTCCATCTAACACCC-3′	81	ca182497, ca095999, ca251048, ca176811, ca076277, cf575388, ca085087, ca205081, ca074129, ca076192
	TST-3	F: 5′-TCAGTGCTGCTCGTGTTTGGT-3′ R: 5′-ATGGCATCACTGGAGGCACTT-3′	130	
	TST-4	F: 5′-AAGTGCCTCCAGTGATGCCA-3′ R: 5′-ACCTGAGGAAACGGAACGCA-3′	200	
*APRT*	APRT-1*	F: 5′-TGACACATTCCCAACCTCAA-3′ R: 5′-ATCTCTCTCCCTGAGTGGCA-3′	119	sb02g033370*
	APRT-2	F: 5′-AGGGAAGTGGTTCGGTGATG-3′ R: 5′-TGATAAAGAGCACATGAACCAACA-3′	74	CA089504, CA089592, CA146761, CA150154, DV640571
*CYC*	CYC-1*	F:5′-CTCATGGAAAACTTACCGGG-3′ R:5′-TGCATCCAGCAAGAAAGTTG-3′	95	sb10g030790*
	CYC-2	F:5′-ACTGATGACATTCCCTTGCCTAT-3′ R:5′-CCGACAGCATAGGCAAGGGA-3′	226	ca176931, ca235106, ca256302, ca101120, bu103284, ca289292, ca074749, ca253051, ca126707
	CYC-3	R:5′-ATAGTCAACCACAGCCAGGGA-3′ F:5′-AACCAACCTACGGTTGCCCA-3′	114	
*PRR*	PRR-1*	F: 5′-GCCAAATTCAGGCAGAAAAG-3′ R: 5′-CACCCTAGGCCTTGTTTCAG-3′	93	sb04g026190*
	PRR-2	F: 5′-GCACCACCCTCCTCTCAGAC-3′ R: 5′-AATGAGCTGGTGGTTGGGGT-3′	261	ca275375, ca297639, ca134882, ca246262, ca245313, ca083303
	PRR-3	F: 5′-ACCAATAGCACCACCCTCCTC-3′ R: 5′-GACGACCCAGCAACCCTCAG-3′	239	
	PRR-4	F: 5′-ACCAATAGCACCACCCTCCTC-3′ R: 5′-GACGACTCAGCAATCCTCAG-3′	239	

Marked with * indicates primer pairs reported in literature [1], while without * are primers redesigned based on the sequences of sugarcane ESTs; P4H: prolyl 4-hydroxylase, ENOL: enolase, TST: thiosulfate sulfur transferase, APRT: anthranilate phosphoribosyl transferase, CYC: cyclin, PRR: pseudo response regulator.

**Table 2.** The standard curve formula, coefficient of determination (*R*^2^) and PCR amplification efficiency (*E*) performed in qPCR assays.

Gene	Primer pairs	Standard curve formula	*R*^2^	*E*

		ROC22 Badila	ROC22 Badila	ROC22 Badila
*P4H*	P4H-1	*y* = −3.212 *x* + 35.610 *y* = −3.051 *x* + 35.328	0.989 0.996	1.048 1.127
	P4H-3	*y* = −3.275 *x* + 37.509 *y* = −3.194 *x* + 36.323	0.998 0.993	1.020 1.056

*APRT*	APRT-1	*y* = −3.174 *x* + 35.962 *y* = −2.918 *x* + 34.326	0.996 0.994	1.066 1.200
	APRT-2	*y* = −3.328 *x* + 37.704 *y* = −3.202 *x* + 39.339	0.998 0.999	0.998 1.053

*CYC*	CYC-1	*y* = −2.321 *x* + 32.405 *y* = −2.218 *x* + 37.622	0.955 0.816	1.697 1.824
	CYC-2	*y* = −3.047 *x* + 34.961 *y* = −3.159 *x* + 37.622	0.992 0.998	1.129 1.073

*TST*	TST-1	*y* = −3.272 *x* + 38.265 *y* = −3.402 *x* + 39.975	0.994 0.999	1.021 0.968
	TST-3	*y* = −3.161 *x* + 37.308 *y* = −3.410 *x* + 38.854	0.999 0.999	1.072 0.965

*ENOL*	ENOL-3	*y* = −3.214 *x* + 37.237 *y* = −3.403 *x* + 44.813	0.999 0.999	1.047 0.967

*PRR*	PRR-1	*y* = −3.185 *x* + 35.916 *y* = −3.514 *x* + 40.872	0.996 0.995	1.061 0.926

*R*^2^, coefficient of determination; *E*, PCR amplification efficiency.

**Table 3.** Estimation of copy number of potential reference primer pairs.

Primer pair	Corresponding gene	*C*t value	Copy number ± SE
	
		ROC 22 Badila	ROC 22 Badila
P4H-3	*P4H*	23.259 22.725	10.47 ± 0.04 C 6.28 ± 0.36 B
ENOL-3	*ENOL*	21.050 24.220	47.68 ± 0.12 A 389.26 ± 1.09 A
TST-1	*TST*	21.689 24.558	51.14 ± 0.21 A 11.76 ± 0.17 B
APRT-2	*APRT*	22.952 24.874	8.39 ± 0.27 C 11.41 ± 0.55 B
CYC-2	*CYC*	21.574 23.678	11.07 ± 0.13 C 9.00 ± 0.53 B
PRR-1	*PRR*	21.143 23.920	19.07 ± 0.23 B 23.06 ± 0.29 B

Capital letters represent significant difference of 1%. Different letters mean significant difference; the same letter indicates no significant difference. SE, standard error.

**Table 4.** Estimation of the copy numbers of three endogenous genes in sugarcane with different genetic background.

Sugarcane genotype	Copy number ± SE		

	*P4H*–P4H-3	*APRT*–APRT-2	*CYC*–CYC-2
Black Cheribon (Yunnan) (*Saccharum officinarum*)	3.58 ± 0.01	4.73 ± 0.47	4.97 ± 0.07
Black Cheribon (Fujian) (*S. officinarum*)	7.32 ± 0.78	9.69 ± 0.16	9.67 ± 0.17
Loethers (*S. officinarum*)	6.37 ± 0.13	9.24 ± 1.03	10.06 ± 0.24
Crystalina (*S. officinarum*)	3.18 ± 0.02	5.03 ± 0.53	4.28 ± 0.28
SES208 (*S. spontaneum*)	7.20 ± 0.66	11.82 ± 0.24	10.95 ± 0.16
LA Purple (*S. officinarum*)	4.44 ± 0.13	7.62 ± 0.95	6.87 ± 0.21
Q117 (*S*. spp. hybrids)	20.81 ± 0.53	22.14 ± 0.27	28.45 ± 0.10
YCE05-179 (*S*. spp. hybrids)	21.51 ± 0.17	13.18 ± 0.12	21.35 ± 0.72

**Table 5.** Coefficient of determination (*R*^2^), PCR amplification efficiency (*E*), copy number and the relative abundance value performed in qPCR assays.

Gene	*R*^2^	*E*	Copy number ± SE		Relative abundance value	
	
			ROC22 Badila		ROC22 Badila	
*P4H*	0.999	1.040	2.91 ± 0.09	2.12 ± 0.06	1.5	1.0
*APRT*	0.999	1.049	2.45 ± 0.09	2.21 ± 0.13	1.0	1.0
*CYC*	0.998	1.047	4.27 ± 0.08	3.05 ± 0.18	2.0	1.5
