# Supplementary material for: Selection of Suitable Endogenous Reference Genes for Relative Copy Number Detection in Sugarcane
Source: Int J Mol Sci. 2014 May 19;15(5):8846–62. doi: 10.3390/ijms15058846 (PMC4057763; doi:10.3390/ijms15058846)
Supplement: Supplementary file 1 [file ijms-15-08846-s001.pdf]

Supplementary Information

Figure S1. The amplification curves, melting curves and standard curves obtained in qPCR assays based on two potential reference primer pairs in Badila (*Saccharum officinarum*). (**a**) P4H-1; (**b**) P4H-3; (**c**) APRT-1; (**d**) APRT-2; (**e**) CYC-1; (**f**) CYC-2; (**g**) TST-1; (**h**) TST-3; (**i**) ENOL-3; (**j**) PRR-1.

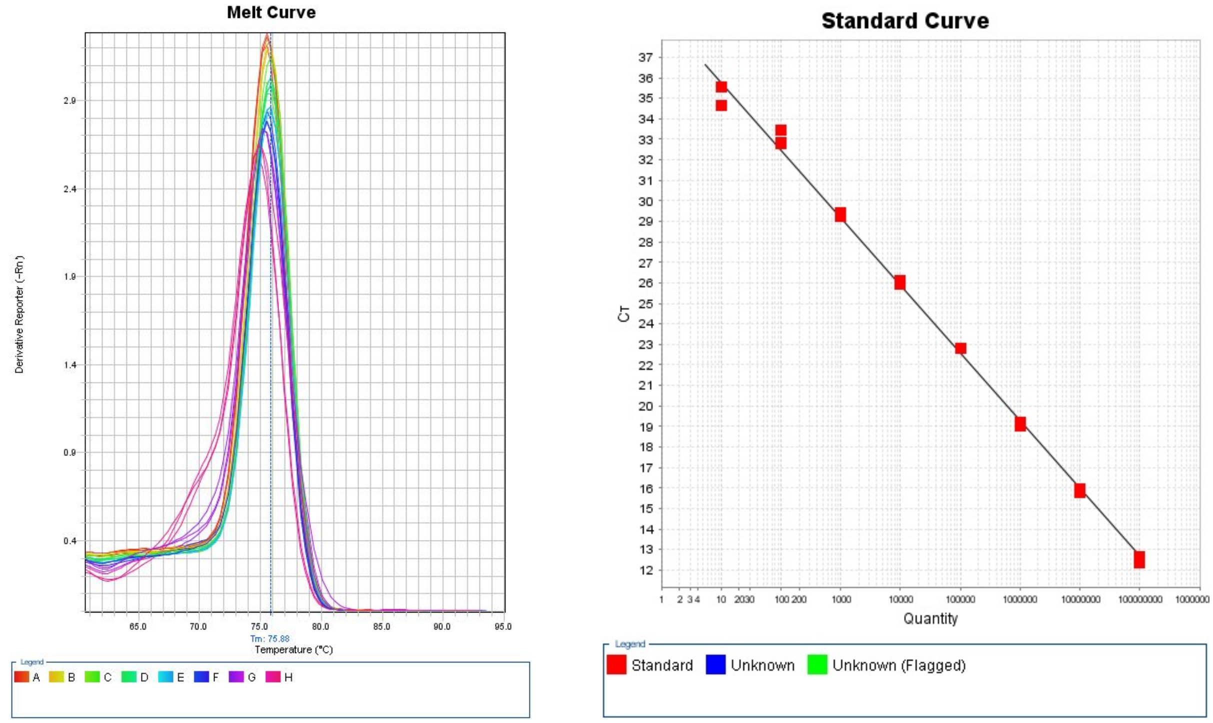

(**a**)

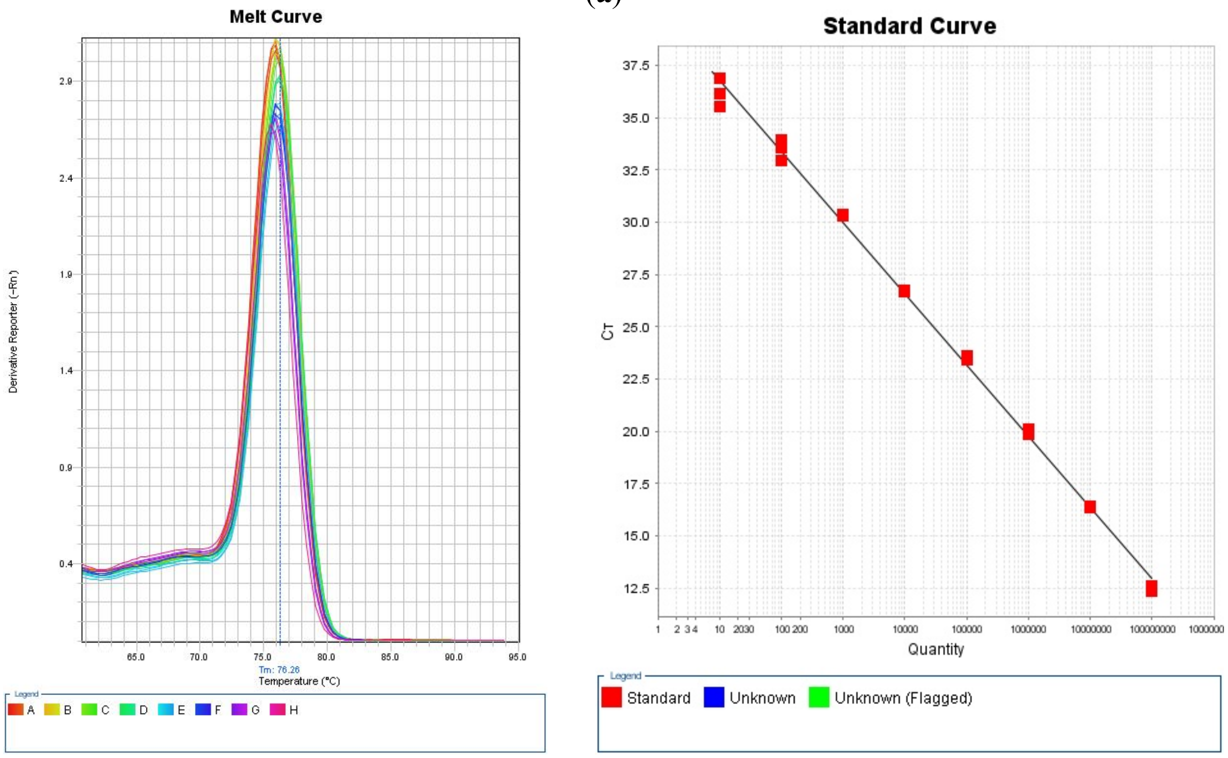

(**b**)

Figure S1. *Cont.*

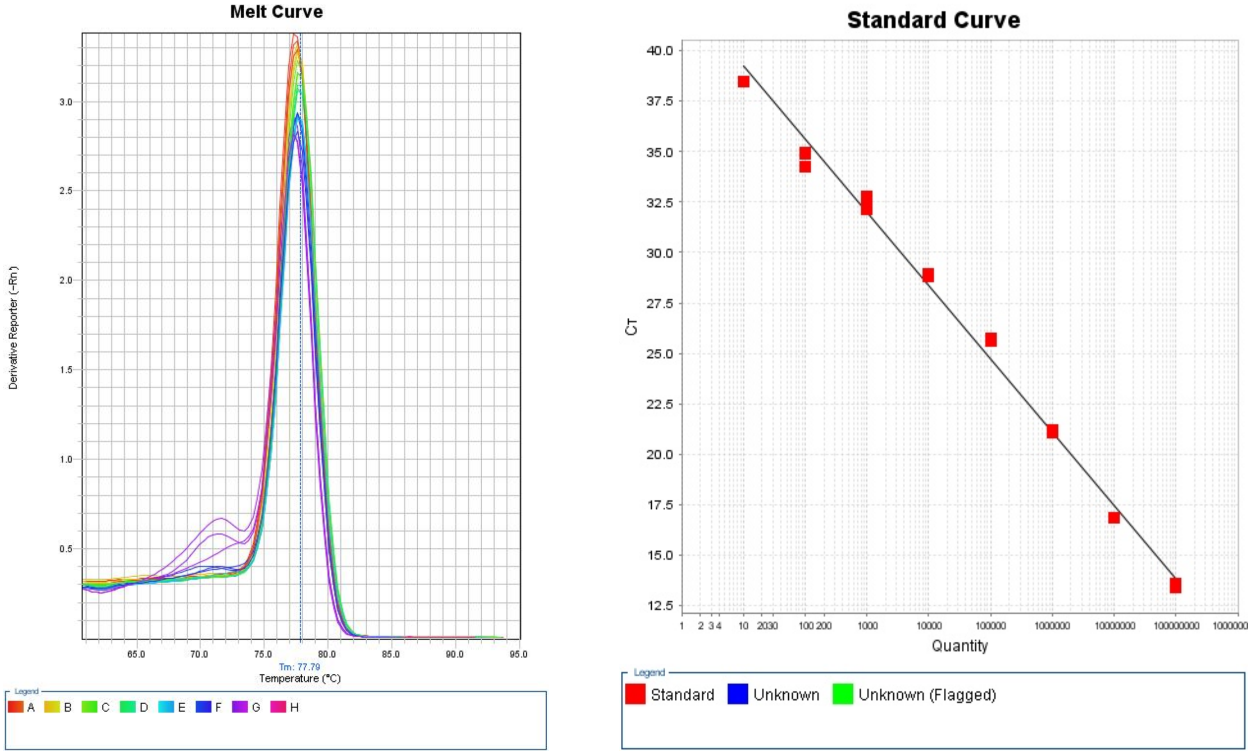

(**c**)

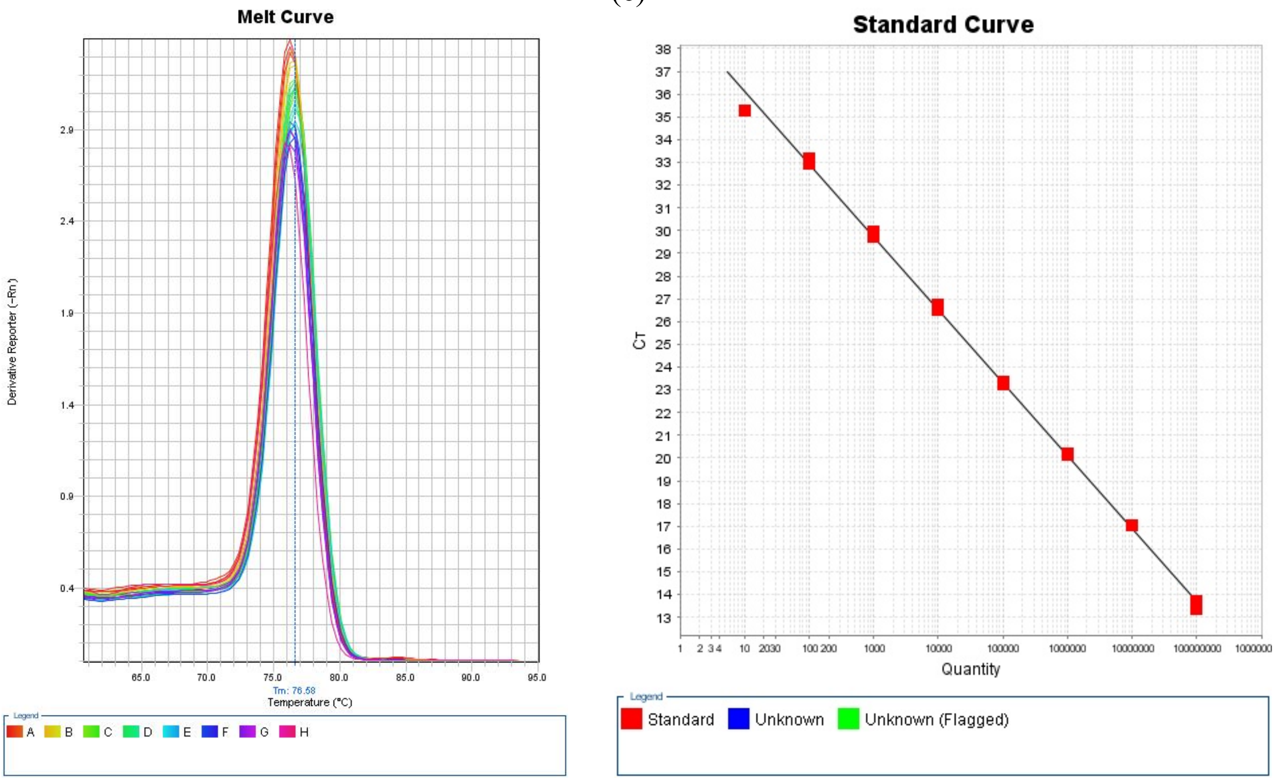

(**d**)

Figure S1. *Cont.*

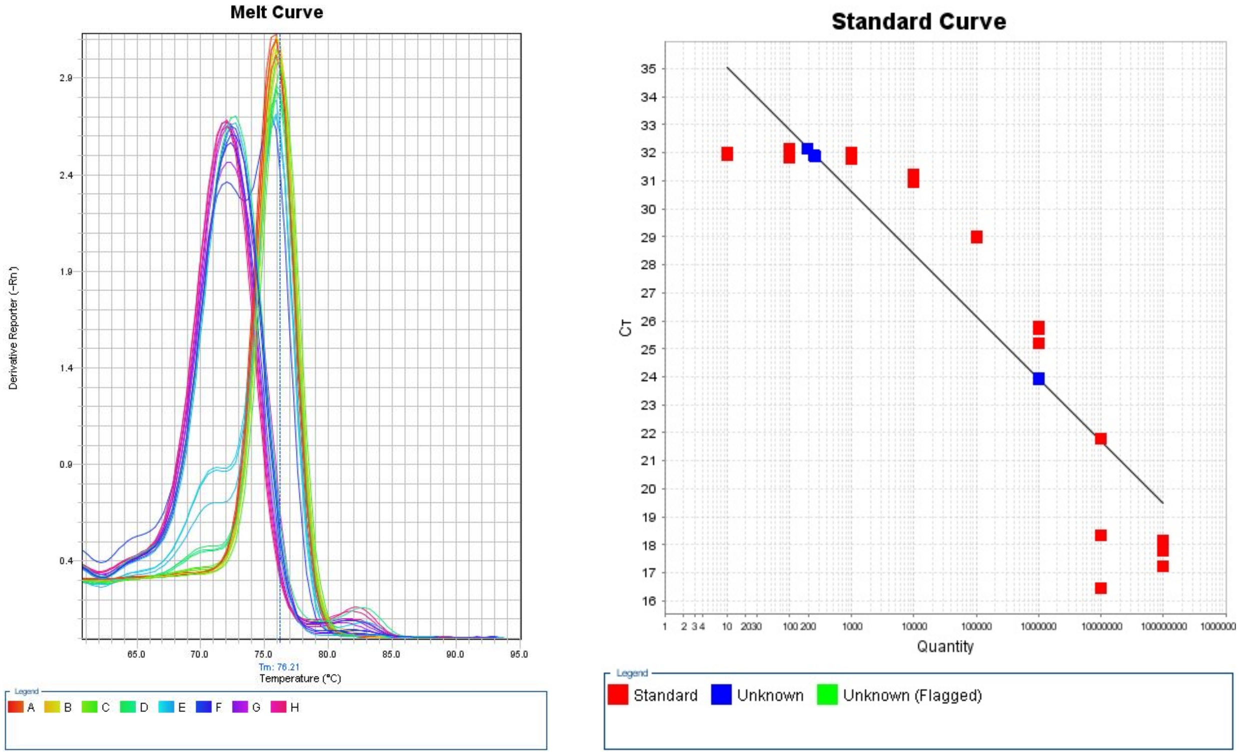

(e)

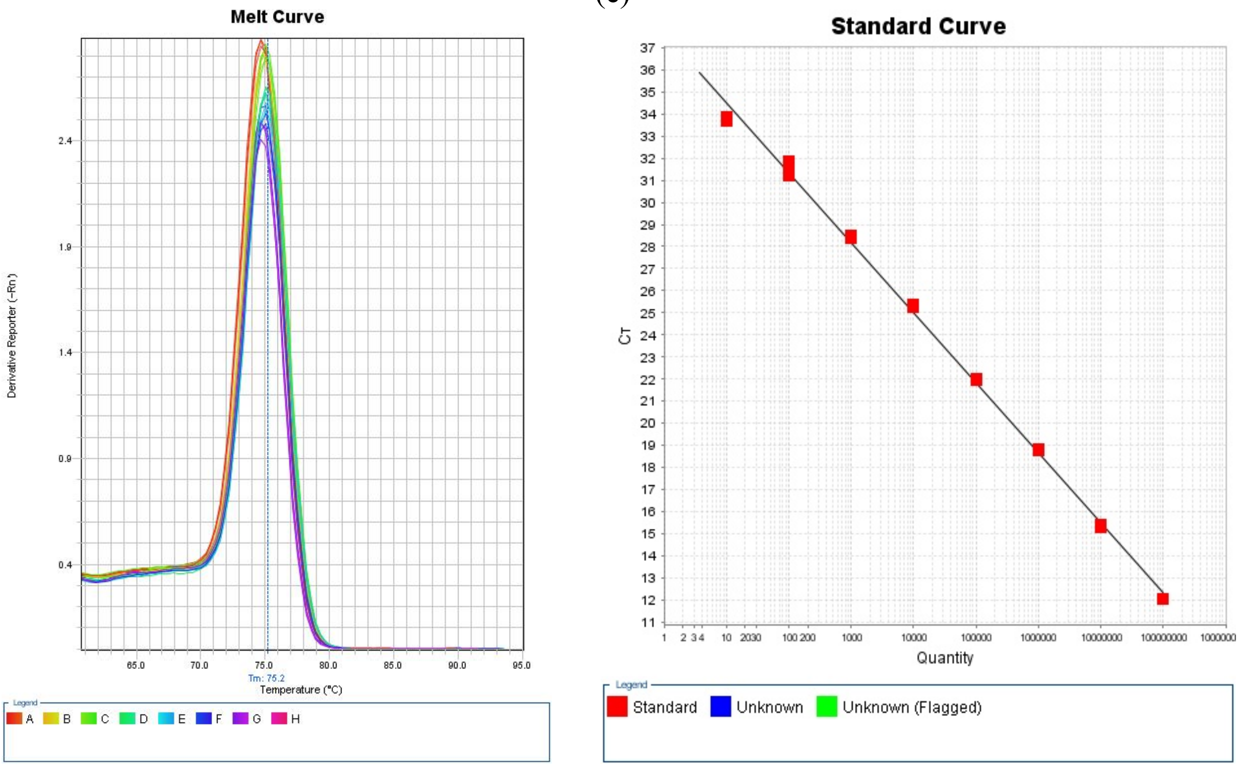

(f)

Figure S1. *Cont.*

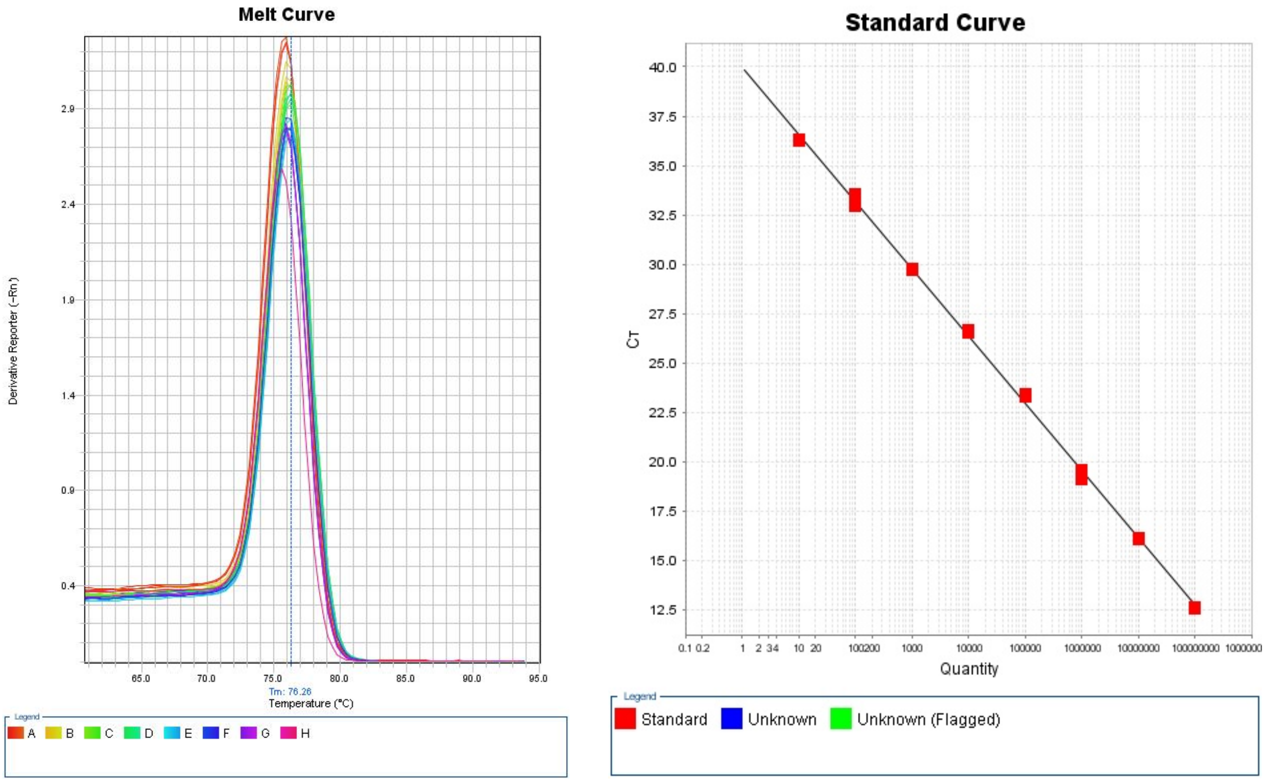

(g)

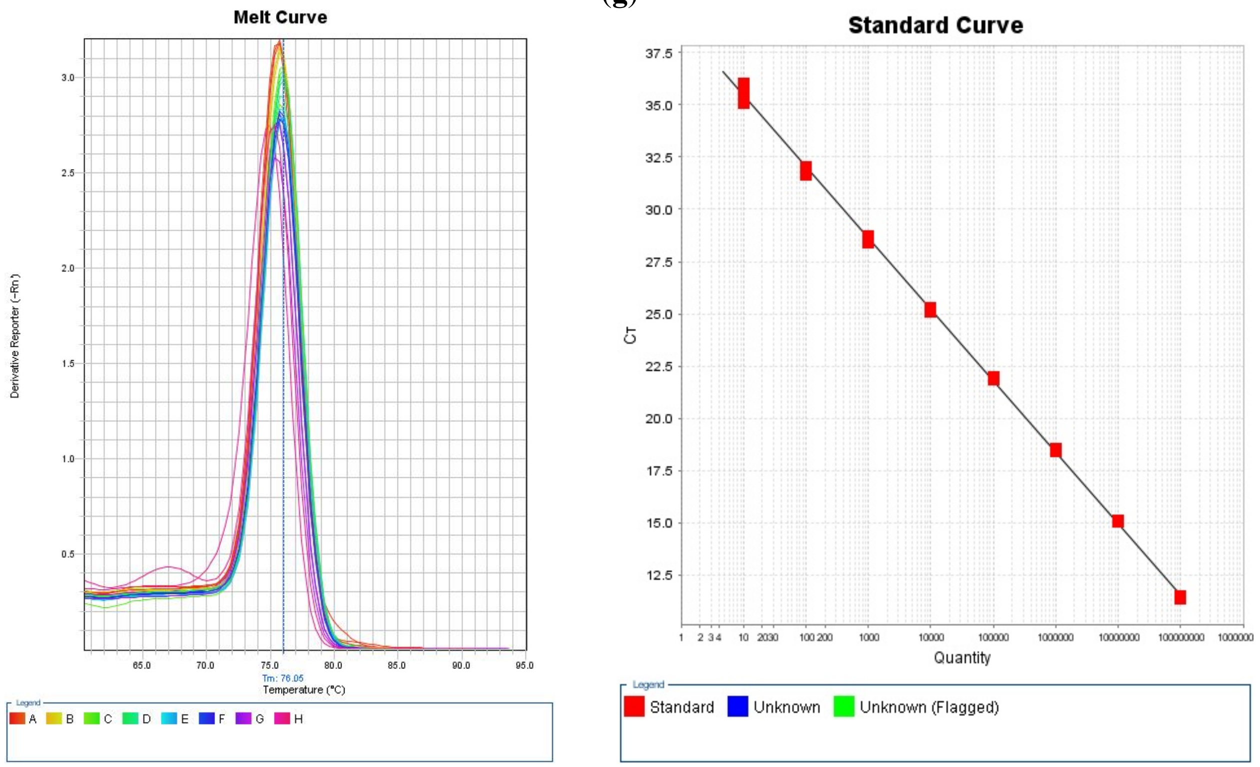

(h)

Figure S1. *Cont.*

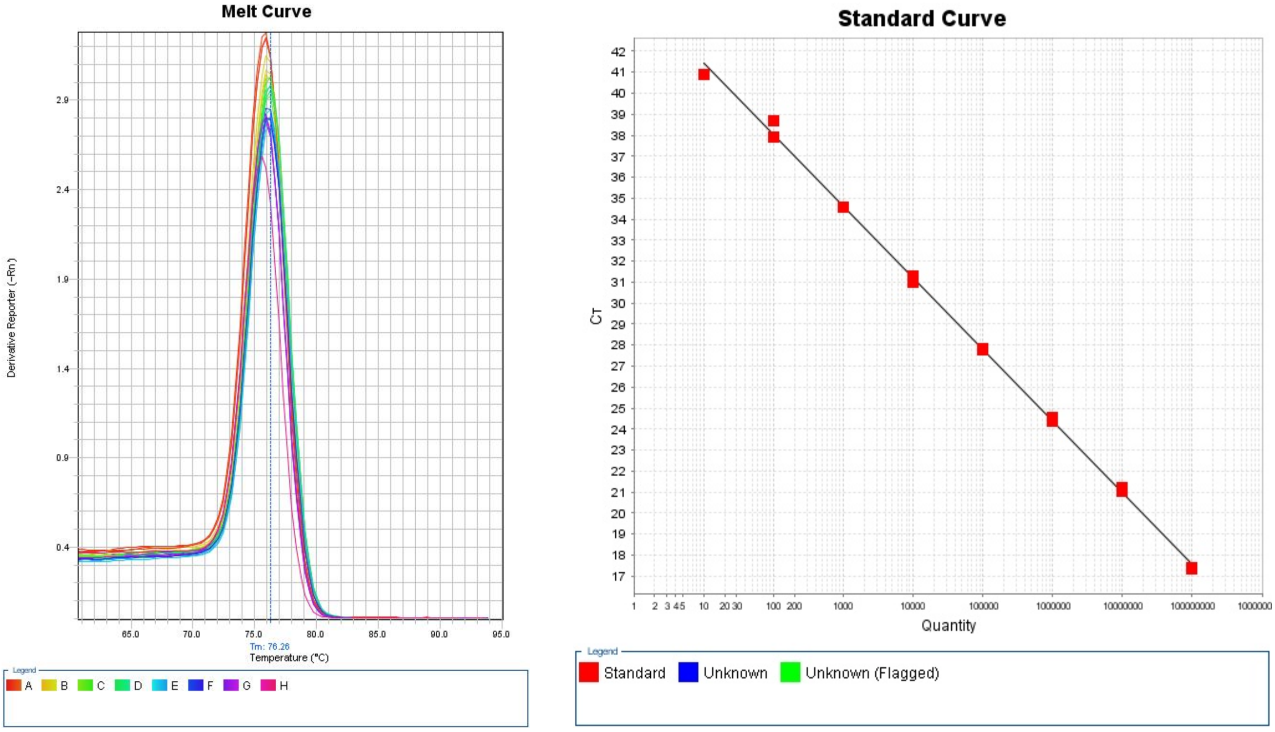

(i)

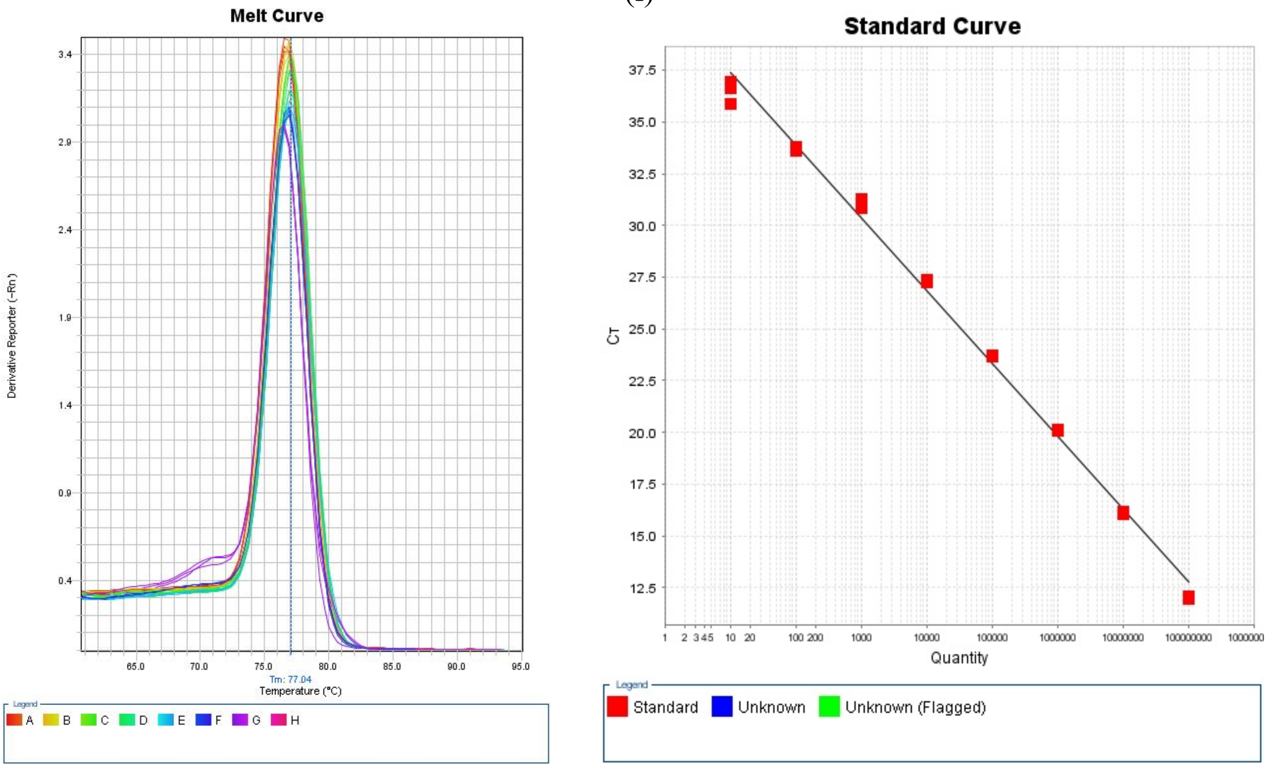

(j)

© 2014 by the authors; licensee MDPI, Basel, Switzerland. This article is an open access article distributed under the terms and conditions of the Creative Commons Attribution license (http://creativecommons.org/licenses/by/3.0/).
